# Characterizing the Interplay Between Autism Spectrum Disorder and Comorbid Medical Conditions: An Integrative Review

**DOI:** 10.3389/fpsyt.2018.00751

**Published:** 2019-01-23

**Authors:** Charlotte Tye, Abigail K. Runicles, Andrew J. O. Whitehouse, Gail A. Alvares

**Affiliations:** ^1^Child & Adolescent Psychiatry, Institute of Psychiatry, Psychology & Neuroscience, King's College London, London, United Kingdom; ^2^Telethon Kids Institute, University of Western Australia, Perth, WA, Australia; ^3^Cooperative Research Centre for Living with Autism (Autism CRC), Brisbane, QLD, Australia

**Keywords:** autism spectrum disorder, comorbidity, epilepsy, gastrointestinal disorders, immune function, sleep

## Abstract

Co-occurring medical disorders and associated physiological abnormalities in individuals with autism spectrum disorder (ASD) may provide insight into causal pathways or underlying biological mechanisms. Here, we review medical conditions that have been repeatedly highlighted as sharing the strongest associations with ASD—epilepsy, sleep, as well as gastrointestinal and immune functioning. We describe within each condition their prevalence, associations with behavior, and evidence for successful treatment. We additionally discuss research aiming to uncover potential aetiological mechanisms. We then consider the potential interaction between each group of conditions and ASD and, based on the available evidence, propose a model that integrates these medical comorbidities in relation to potential shared aetiological mechanisms. Future research should aim to systematically examine the interactions between these physiological systems, rather than considering these in isolation, using robust and sensitive biomarkers across an individual's development. A consideration of the overlap between medical conditions and ASD may aid in defining biological subtypes within ASD and in the development of specific targeted interventions.

## Introduction

Autism spectrum disorders (ASD) are a group of complex and heterogeneous developmental conditions characterized by reduced social interaction and communication, as well as restricted range of interests and/or stereotypic behaviors (1, 2). Many of the core cognitive and behavioral symptoms associated with ASD are thought to arise from dysfunction of the central nervous system (3). However, accumulating and converging evidence across several areas of medicine have strongly emphasized comorbid medical conditions and associated peripheral/central physiological abnormalities in children with ASD as providing potential clues to additional etiological factors. Epidemiological evidence indicates that medical disorders are more prevalent in children with ASDs compared to children in the general population (4), with further evidence for specific clusters of medical conditions in ASD (5). Whilst multisystem dysfunction across a range of organs and physiological systems has been proposed in other brain-based conditions (including neurodegenerative disorders, schizophrenia, bipolar disorder and depression), the elevated incidence of medical conditions and associated markers of dysfunction appear to be particularly perturbed in ASD (6). These medical conditions, and accompanying disabilities, can have significant impacts on broader development outcomes, social functioning, and education/employment outcomes. Despite the high prevalence of medical comorbidities in this population, many of these conditions are not routinely screened for in ASD evaluations (7, 8). This may reflect variability in the symptoms expressed by individuals with varying language capacity as well as the ability of health professionals to recognize behavioral symptoms that may be better explained by other medical conditions. In addition to better medical treatment, the increasing understanding of systemic abnormalities in ASD may provide clues to etiological factors as well as identifying biological pathways for more targeted and effective treatments.

Prevalence estimates of medical comorbidities vary greatly depending on the population studied. One of the largest studies of hospital records of children and young adults with ASD in the US found that prevalence estimates of a range of co-morbid disorders, particularly gastrointestinal and seizure disorders, greatly exceeded that compared to the general hospital population (4). Other studies have highlighted greater prevalence of abnormal neurological findings and clinical neuropathology in children with ASD (9). Strikingly, reports indicate elevated mortality ratios of up to 2.4 in ASD (10–12), which is thought to be due to largely due to complications arising from comorbid medical conditions (13). Put another way, premature mortality in individuals with ASDs is over twice the rate of that experienced by the general population (12, 13). Although prevalence estimates for some conditions are higher in studies with smaller samples, the overall evidence suggests that at least 10% of individuals with ASD present with comorbid medical symptoms that require formal medical evaluation (4). This has significant implications for subsequent development, prognosis and treatment plans for individuals and families as well as the additional pressures comorbidities place on health care and disability systems to provide adequate and necessary supports across the lifespan.

**Figure 1:**
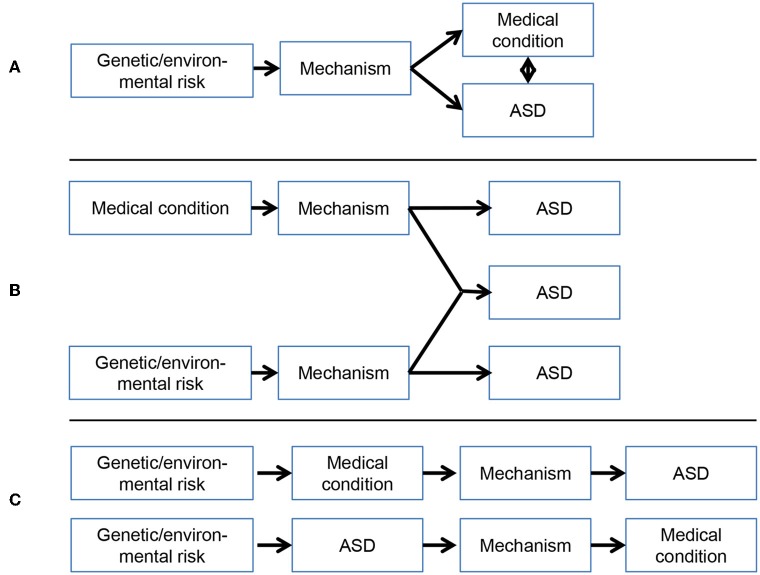
Possible models of the association between medical conditions and ASD: **(A)** Overlap between medical conditions and ASD arises from a *common* mechanism; **(B)** Overlap between medical conditions and ASD arises from the *independent* pathways or *cumulative* impact of impairments in two or more developmental pathways (possibly subgroups of individuals); **(C)** Overlap between medical conditions and ASD arises from the *effect of medical abnormalities* on underling mechanisms, or vice versa. These models are not mutually exclusive and more than one pathway may be involved.

There have been several reviews and empirical studies focusing on specific medical, [e.g., (14–17)], behavioral [e.g., (18–20)], and genetic [e.g., (21, 22)] comorbidities of ASD. Here, we review medical disorders that have been repeatedly highlighted as sharing the strongest associations with ASD—epilepsy, sleep problems, gastrointestinal disorders and immune dysfunction—with the purpose of integrating mechanistic theories of their overlap. For each domain, we describe the prevalence and associations with behavior and cognition in ASD, before moving on to focus on work aiming to uncover shared aetiological mechanisms. A proposed mechanistic model integrating ASD and these medical comorbidities is then presented. We argue that a consideration of overlap between medical problems and ASD will provide insight into shared mechanisms and implications for treatment, based on proposed models of comorbidity (Figure 1).

## Meta-Synthesis of Theories Underlying Common Medical Comorbidities of ASD

### Epilepsy

#### Prevalence

Epilepsy, defined as two unprovoked seizures of any type, can be extended out to include multiple disorders with various etiologies, pathophysiology and outcomes (23). Prevalence of epilepsy in the general population is between 1 and 2%, whilst general estimates suggest a prevalence of ~25–30% in individuals with ASD by adolescence (24, 25). In particular, two peak periods of epilepsy onset have been described in ASD—one in early childhood and a second in adolescence (26, 27), although prospective longitudinal studies have failed to replicate this bimodal distribution (28). Rates of ASD are higher in certain genetic disorders; for example, 47.4% of individuals with Dravet syndrome meet criteria for ASD, with the main seizure type being focal seizures manifesting in clusters (29). Whilst rates of ASD in tuberous sclerosis complex (TSC) are thought to approach 60%, individuals with TSC often experience different types of seizures (30, 31). Even in the absence of diagnosed epilepsy, there is considerable debate concerning the significance of abnormal electroencephalography (EEG) findings observed in ASD not associated with clinical seizure activity (24). A significant proportion of individuals with ASD display significant EEG paroxysmal abnormalities during sleep without the presence of clinical seizures, with reports as high as 60% (32–34). Retrospective studies indicate similar rates of cognitive impairment and cerebral lesions in ASD patients with abnormal EEGs with and without epilepsy (27). Due to this debate, clinical EEGs are not generally recommended as routine practice for children with ASD unless seizure activity is suspected.

All seizure types appear to be associated with ASD but vary in prevalence depending on the population studied (see Table 1). In a Swedish study, the most prevalent seizures in ASD were complex partial, atypical absence, myoclonic, and tonic-clonic seizures (35); by comparison, an American study reported that generalized tonic-clonic and atypical absence seizures were the most common in ASD (36). Some more recent studies argue that complex partial seizures are most prevalent in ASD (27, 34, 37). Clinically, this latter seizure type is particularly significant as some symptoms of complex partial seizures may be difficult to differentiate from common associated behaviors in ASD, such as not responding to calling name or repetitive movements. Of significance for clinical intervention, it has also been reported that treatment-resistant epilepsy is also of particularly high prevalence in ASD (38). One of the most severe forms of comorbid epilepsy in ASD is epileptic encephalopathy, a process whereby the epileptic activity contributes to severe cognitive and behavioral impairments above and beyond the underlying pathology alone (39, 40). It is characterized by intractable seizures as well as frequent ictal or interictal epileptiform activity (39), which may be idiopathic or syndromic. Infants with epileptic encephalopathy are at a higher risk for an ASD diagnosis and lasting cognitive impairments (41). In particular, 19.9% of children with infantile spasms will have ASD (29).

**Table 1:** Overview of studies examining specific seizure types in individuals with ASD.

**Study**	**Sample size**	**Prevalence**	**Subtypes**
Steffenburg et al. (35)	*N* = 90, 24 ASD, 53 with at least 1 psychiatric diagnosis.	59% at least 1 psychiatric diagnosis, 27% ASD, 11% had ASD-like condition.	Complex partial, atypical absence, myoclonic, tonic clonic.
Tuchman et al. (36)	*N* = 314 ASD, *N* = 237 dysphasic non-ASD.	14% of ASD and 8% of dysphasic had epilepsy.	Generalized tonic-clonic and atypical absence seizures.
Matsuo et al. (37)	*N* = 519 with epilepsy, *N* = 79 ASD.	15.2% of sample with epilepsy had ASD.	Most frequent type—complex partial seizures.
Yasuhara (34)	*N* = 1,014 ASD.	37% had epilepsy.	Most frequent type—complex partial seizures.
Parmeggiani et al. (27)	*N* = 345 ASD.	24.9% epilepsy, 45.5% EEG paroxysmal abnormalities.	Most frequent type—complex partial seizures.

Of concern, is the increased 2-fold higher mortality rate in individuals with comorbid ASD and epilepsy compared to the general population, which is higher still in females with these comorbidities (11, 13, 42). These studies describe sudden unexpected death due to epilepsy at a higher frequency than expected, indicating that these individuals are likely to require increased medical monitoring to prevent avoidable death over their lifespan.

#### Associations With Behavior and Cognition in ASD

Research has suggested that there is a relationship between specific symptoms and epilepsy in individuals with ASD. Turk et al. (43) found that a co-morbid diagnosis of ASD and epilepsy led to greater difficulties in in social interactions with peers and unusual eye contact. Those with epilepsy also received an ASD diagnosis later than those who were not diagnosed with epilepsy. Conversely, other studies have found that increased frequency of epilepsy has been associated with an earlier age of ASD diagnosis, higher rates of repetitive object use, and greater unusual sensory interests (44). Viscidi et al. (45) completed a large-scale study showing that children with both epilepsy and ASD displayed significantly more maladaptive behaviors linked to ASD. This included a decrease in scores on social cognition, communication and motivation when compared to ASD patients without epilepsy. Participants with both epilepsy and ASD also display higher levels of self-injurious, compulsive and sameness behaviors. Finally, a meta-analysis of social cognition in epilepsy and autism and found that rates of impaired facial recognition and theory of mind were higher in patients with epilepsy and ASD compared to controls (46). These studies suggest that there is a link between epilepsy and ASD symptoms and cognitive correlates. However, the close relationship with IQ suggests a dynamic interplay between ASD symptoms, and subsequent diagnostic age, IQ, and epilepsy.

Evidence from meta-analyses confirms an overall greater risk for epilepsy in children with ASD and intellectual disability (21.5%) compared to those without comorbid intellectual disability (8%) (47), as well as an increased risk in children with ASD and intellectual disability after the age of 12 years (48). This implies that the neurobiological mechanisms underpinning common associations between epilepsy and ASD may be derived from these subgroups within ASD. Other researchers have also pointed to the relationship between behavioral regression in ASD and greater incidence of epilepsy (49–51). Berg and Pliophys (52) suggest that the role of intellectual disability is so vital that it could explain most, if not all of the relationship between epilepsy and ASD. In a case study of TSC, the child's development was typical until 21 months of age at which seizure onset occurred; by 24 months of age the child met criteria for both ASD and intellectual disability (53). Taken together, this suggests that there is a dynamic interplay between seizures, intellectual disability and ASD with the onset of seizures playing a significant role in developmental regression and ASD diagnostic symptoms.

A diagnosis of comorbid epilepsy and ASD is a negative prognostic indicator, associated with greater risk of poorer outcomes (54). Children with both conditions report lower quality of life, score lower on social maturity scales, and exhibit higher use of psychotropic medications (26, 55, 56), in addition to poorer social outcomes (43). A large prospective follow-up study found significantly higher levels of cognitive impairments in adults followed from childhood, with seizure frequency reported to have the most significant impact on individual functioning (28). Even in children with ASD but without diagnosed epilepsy, EEG abnormalities and seizures have been associated with greater reports of aberrant behavior [(57); see Table 2].

**Table 2:** Overview of studies examining behavior and cognitive differences in individuals with ASD and epilepsy.

**Study**	**Sample size**	**Features in ASD + epilepsy compared to ASD**
Turk et al. (43)	*N* = 60 ASD + epilepsy, *N* = 60 ASD.	Higher incidence of motor difficulties, developmental delays & challenging behaviors.
Cuccaro et al. (44)	*N* = 577 ASD, *N* = 64 ASD + epilepsy.	Higher rates of repetitive object use and unusual sensory interests.
Viscidi et al. (45)	*N* = 2,645 ASD, *N* = 139 ASD + epilepsy.	Higher rates of irritability (20% higher) and hyperactivity (24% higher).

#### Treatment Effects

As epilepsy may contribute to elevated mortality rates in ASD, appropriate detection and treatment of elevated seizure activity in individuals with ASD is a clear and urgent treatment need [(48); see Table 3]. Treatment of children with both epilepsy and ASD is based on guidelines aimed at treating childhood epilepsy with anti-epileptic drugs (AEDs), chosen based on seizure type and response to medication. Research into treatments that best control seizures in children with ASD is critical, as some children with ASD appear to be more sensitive to the side effects of AEDs (24). When epileptiform activity is present, therapeutic interventions to reduce seizure activity may also improve language outcomes and ASD symptoms [reviewed in (62)]. Valproic acid, lamotrigine and Levetiacetam are the mostly highly rated AEDs in terms of effectiveness and tolerability in ASD, with valproic acid also found to improve core ASD symptoms (58). While alternative treatments, such as diets (e.g., ketogenic, modified Atkin's or gluten and casein free), have shown some efficacy for treating epilepsy, their suitability and safety for individuals with ASD require further study. Recent treatment advances for syndromic ASD may have particular relevance in improving either or both seizures and symptoms associated with ASD. For example, mTOR inhibitor treatment (Everolimus), associated with reduction in cell proliferation, angiogenesis and glucose uptake, has shown efficacy for some of the physical manifestations of TSC (63). In a case series, treatment with Everolimus was associated with a reduction in the severity and frequency of seizures, as well as improvements in repetitive behavior and social contact (59), although a recent study showed limited effect of everolimus on neuropsychiatric features in children (60). Furthermore, vigabatrin is recommended for children with infantile spasms and TSC, linked to improved later outcomes, including a decrease in adverse cognitive and behavioral outcomes such as ASD (61). Such treatment effects may help to explain the underlying pathophysiology and interaction between genetic mutations, epilepsy and ASD, for example, through normalization of altered brain activity and excitatory/inhibitory balance (see section Aetiological Mechanisms).

**Table 3:** An overview of different therapeutic approaches to treating epileptic symptoms in ASD.

**Studies**	**Therapeutic approach**	**Benefits of therapeutic approach**	**Caveats of therapeutic approach**
Frye et al. (58)	Anti-epileptic drugs (AEDs; e.g., Valproic acid, lamotrigine, levetiracetam and ethosuximide)	Improvement in seizures and limited impact on other clinical factors	Rate of side effects higher for AEDs compared to non-AED treatments
Frye et al. (58)	Ketogenic diet	Improvement in seizures, favorable effects on sleep, communication, behavior, attention and moodLow incidence of adverse effects	Can result in severe acidosis
Kilincaslan et al. (59) Krueger et al. (60)	mTOR inhibitor (Everolimus)	Reduction in severity and frequency of seizures, improvements in repetitive behavior and social contact (59)	Limited effect on neuropsychiatric features (60)
Bombardieri et al. (61)	Vigabatrin	Decrease in adverse cognitive and behavioral outcomes	Only indicated in patient with TSC and infantile spasms

#### Aetiological Mechanisms

Seizure disorders can be due to many causes, including acute or chronic focal or diffuse brain pathology, genetic mutations, metabolic causes, or idiopathic reasons; as such, attempts to identify common causal pathways between ASD and epilepsy have proven to be a complex endeavor. While a number of reports have suggested common neuropathology in children with ASD and epilepsy (64), the vast majority of evidence suggests that epilepsy is not causal to the development of ASD, although the notable exception may be in the case of infants with an epileptic encephalopathy (see section Prevalence). It has been hypothesized that comorbidity of these conditions reflects shared neurodevelopmental pathways or common etiopathologies (65), such as abnormalities in synaptic plasticity and excitatory/inhibitory imbalance early in development and/or shared multiple genome variants (25, 40).

Many candidate genes associated with ASD and epilepsy are involved in synaptic formation and maintenance (e.g., NRXN1, CNTNAP2) and GABAergic neurotransmission [e.g., ARX, Mecp2; for review see (66)]. The possibility of common genes is especially pertinent for those with intellectual disability. Recurrent structural abnormalities are shown at 15q13.3, 16p13.11, and 16p13.3 in patients with epilepsy, which overlap with genomic hotspots reported in ASD and ID (47, 67, 68). Likewise, copy number variations (CNVs) identified in patients with epileptic encephalopathies (Landau-Kleffner syndrome and continuous spike-wave during slow-wave sleep syndrome) corresponded to genomic regions or genes associated with ASD or related behaviors, particularly those encoding cell adhesion proteins (69).

The association between genetic disorders, epilepsy and ASD could provide a further indication of the aetiological mechanisms underlying ASD and epilepsy (40). Genetic disorders associated with epilepsy, such as TSC, Dravet syndrome and Angelman syndrome, have been linked to higher rates of ASD (30, 70–72). For example, TSC is caused by a mutation of TSC1 or TSC2 that disrupt the mTOR pathway and is characterized by multisystem growths of tumor-like lesions called hamartomas, which can affect a wide-range of bodily systems. Cortical tubers and peritubular cortex act as epileptogenic foci which increase the risk of epilepsy up to 90% and are linked to intellectual impairment and behavioral disturbances including ASD (70, 73). Likewise, Dravet syndrome (severe myoclonic epilepsy in infancy) is often caused by mutations in the SCN1A gene, which regulates movement of sodium ions, helping to propagate electrical signals along neurons (74). Dravet syndrome is associated with prolonged refractory seizures within the first year of life and later cognitive impairment, motor deficits and behavioral disorders such as ASD (71). Duplications on chromosome 15q11.3-q13.1 (Dup15q syndrome) lead to overexpression of several genes, including those involved in GABA transmission and are associated with high risk for early onset epilepsy, ASD and intellectual disability (75). The association between ASD and epilepsy within genetic syndromes may help to identify a shared etiology, whereby disruption downstream of the genetic mutation affects both epileptogenesis and behavior [for review see (76)]. The genes implicated in several of these syndromes are associated with synaptic function. The diverse genetic etiologies identified may therefore converge by altering excitatory/inhibitory balance in the cerebral cortex, due to defects in GABAergic fibers of GABA-receptor function (77).

Despite many common associations found in both disorders, there is a limited amount of evidence investigating shared environmental and/or neuroinflammation pathways leading to comorbid epilepsy and ASD, for example advanced maternal/paternal age (78) or increased activation of astroglia/microglia in children with ASD (79) or epilepsy (80).

#### Summary

The well-documented overlap between ASD and epilepsy points toward shared aetiological mechanisms. Current findings do not suggest a causative role for epilepsy and abnormal epileptiform activity in the development of ASD; rather, presence of comorbid conditions predicts poorer prognostic outcome in individuals, and as such, indicates need for greater monitoring and interventions for associated factors. Research to date has not yet been able to fully determine the anatomical or molecular causes of why these conditions converge, as it is difficult to distinguish the effects of the underlying pathology from the neurological effects of the seizures themselves (25).

### Sleep Problems

#### Prevalence

Sleep problems occur in a significantly higher proportion of children with ASD compared to typically developing children and children with other developmental delays (81). Prevalence estimates range between 50 and 80% as compared with 9–50% of typically developing children (81–84). These sleep disturbances not only affect daytime functioning but impact on the quality of life of the whole family (85).

A range of sleep disorders may be present in children with ASD, including insomnia (including difficulties with sleep initiation, duration, consolidation, or sleep quality, bedtime resistance, night awakenings, or inability to sleep independently), sleep disordered breathing (that is, disorders related to airway obstruction, including obstructive sleep apnoea), parasomnias (including nightmares, wake screaming, complex movements, and dreams), and sleep related movement disorders (for example, rhythmical movement disorder and restless legs syndrome). Using parental reports, Goldman and colleagues found that younger children with ASD experienced more sleep anxiety, bedtime resistance, night wakefulness, and parasomnias; adolescents, however, tended to have more difficulty falling asleep, getting enough sleep, and experiencing daytime sleepiness (86). Actigraphy data confirms this, with children with ASD experiencing greater latency to fall asleep, longer periods of awakenings, and more night-time activity compared to typically developing children (87). Data from overnight polysomnography also demonstrates shorter sleep times and lower rapid eye movement (REM) sleep in children with ASD (88). Overall, insomnia (difficulty falling asleep and staying asleep) appears to be the most reliably observed phenomena to characterize sleep disturbances across the spectrum [(89–91); see Table 4].

**Table 4:** An overview of sleep difficulties in individuals with ASD.

**Source**	**Sample size**	**Methodology**	**Prevalence**	**Sleep disturbance**
Goldman et al. (86)	*N* = 1,859 ASD	Parent-report questionnaire	67.3% categorized as good sleepers, 31.5% as bad sleepers	Younger children had sleep anxiety bedtime resistance, night waking and parasomnias. Adolescents had problems with falling asleep, getting enough consistently and daytime sleepiness
Souders et al. (87)	*N* = 59 ASD, *N* = 40 Controls	Actigraphy	66% in ASD group had moderate sleep disturbances compared to 45% mild sleep disturbance in controls	Behavioral insomnia sleep-onset type
Buckley et al. (88)	*N* = 60 ASD, *N* = 15 Controls	Overnight polysomnographic recording	–	Shorter total sleep time and smaller REM sleep percentage
Baker et al. (90)	*N* = 34 ASD, *N* = 27 Controls	Sleep questionnaire, sleep diary, and actigraphy	Three times more likely to report sleep problems compared to controls	Decreased sleep efficiency and fatigue

#### Associations With Behavior and Cognition

Sleep has a vital role to play in child development as it serves multiple functions, such as energy conservation, brain growth, cognition and memory consolidation (15). The relationship between behavior in ASD and sleep dysfunction are likely to be bi-directional; whilst challenging daytime behaviors and associated comorbid conditions (e.g., attention deficit hyperactivity disorder (ADHD), anxiety and depression) contribute to sleep difficulties (see also section Treatment Effects), inefficient sleep can exacerbate or promote ASD behaviors. For example, sleep disorders are predictive of symptom severity in children with ASD (92, 93), including the level of social interaction difficulties (94). Different sleep problems appear to be differentially related to behavioral difficulties. For example, decreased sleep duration (and associated daytime sleepiness) has been associated with increased severity of core ASD symptoms such as repetitive behaviors and social communication difficulties, as well as pronunciation of more specific features such as failure to develop peer relationships and adherence to non-functional routines or rituals (95). Sleep onset delay is associated with stereotyped behaviors and social interaction deficits, but not communication deficits (96), while parasomnias has been linked to symptom severity, communication problems and an increased in stereotyped behaviors (96). Alterations in specific sleep stages may also be associated with different ASD phenotypes; prolonged REM latency has been associated with regression (97). Other maladaptive behaviors, such as self-injury, tantrums and aggression, are associated with shorter sleep durations in ASD (15), and children with ASD with poorer sleep quality also have higher rates of internalizing and externalizing behavioral disorders and lower levels of adaptive functioning (98, 99).

Sleep disorders may be caused by core ASD symptoms; for example, children may have reduced sensitivity to environmental cues that help signal the sleep/wake circadian systems, perseverate on activities or thoughts that may interfere with sleep onset or promote nocturnal awakenings, have limited communication to understand parental expectations for bedtime, or have more challenging behaviors such as hyperactivity or environmental hypersensitivities that may preclude ability to settle down to sleep (100). However, comorbidities such as anxiety or depression may also contribute; in typically developing children, sleep disorders are often related to anxiety and depression (15, 101). Children with ASD are especially vulnerable to co-occurring psychopathologies with prevalence rates ranging from 25 to 70% within the ASD population (18). For example, insomnia can be a consequence of elevated levels of anxiety in individuals with ASD (102), and higher percentage of time spent in REM sleep is associated with greater internalizing behavior in ASD (103). Interestingly, melatonin treatment may alleviate symptoms of both insomnia and anxiety in ASD (104), supporting a shared etiology.

Rates of co-occurring ASD and ADHD are also high, with the prevalence of ADHD symptoms in individuals with ASD ranging from 40 to 70% (105). The key symptoms of ADHD, inattention and hyperactivity, can have an impact on both an individual's ASD symptoms and thus the development of a sleep disorder (85). It has been hypothesized that symptoms of sleeplessness do not always manifest as sleepiness but as overactivity, which at extreme levels can be classified as hyperactivity (85). Interestingly research has shown that melatonin treatment can also have a positive effect on reducing both sleep disorder and hyperactivity (104), suggesting that insufficient sleep may result in behavioral symptoms often seen in patients with co-morbid ASD and ADHD.

#### Treatment Effects

Potential treatments for sleep disturbances range from behavioral to pharmacological, with the goal to not only improve sleep quality and daytime functioning but also reduce caregiver stress (106). Although a number of behavioral and medical sleep interventions are available, only melatonin appears to have been systematically investigated for its efficacy in ASD (107, 108). The summary evidence for melatonin supplementation for sleep problems in ASD suggests significantly improved sleep duration and sleep onset latency compared to placebo, and significantly improved daytime behaviors, with minimal side effects (107, 108). Because sleep deprivation can contribute to emotional reactivity and interpretation of nonverbal social cues (109, 110), sleep disturbances and daytime sleepiness may also contribute to efficacy of daytime behavioral interventions or impact on educational outcomes.

Conversely, it has also been argued that medications often prescribed to treat symptoms such as anxiety in individuals with ASD may negatively influence sleep. For example, it has been suggested that anti-psychotics and serotonin reuptake inhibitors (SSRIs) may disrupt the sleep cycle and thus have a role to play in sleep disorder (111). Some medications also increase risk of metabolic side effects like obesity (112); rates of overweight and obesity are increased in children with ASD, particularly those with co-occurring sleep disorders (113). The mechanisms and directional impact of sleep problems and treatment in ASD therefore remain complex.

#### Aetiological Mechanisms

Development of a circadian rhythm has a vital role to play in the development of a competent sleep cycle, controlling different biological rhythms within a 24-h period and modified by both internal and external factors. It is thought that this rhythm regulates both biological and behavioral functions and how an individual can anticipate and adapt to environmental changes (114). When the circadian cycle is dysregulated by underlying alterations in neurophysiological and neurochemistry, an individual can be vulnerable to sleep disorders and physiological disturbances (15, 114). Neurochemistry factors involved in normal sleep include neurotransmitters such as GABA (115), serotonin, and melatonin (116).

**Figure 2:**
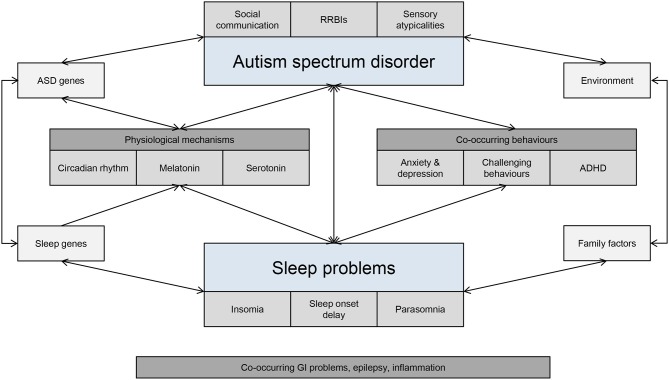
Interdependent bidirectional associations between ASD and sleep problems. GI, gastrointestinal; RRBIs, restricted and repetitive behaviours and interest.

Genes known to be associated with the human circadian clock have an important role to play in controlling sleep phase and duration (117) and have a widespread physiological effect on mood, cognition and reward-related behaviors. Mutations in clock genes have been implicated in ASD, thus resulting in the dysregulation of the circadian cycle (114, 118). For example, mutations in the Per1 and NPAS2 clock genes are associated with ASD and related to the morningness-eveningness phenotype (119, 120), and missense changes in six clock genes have been identified in individuals with ASD and sleep problems (118). In addition, the SCN controls the sleep-wake cycle by stimulating the pineal gland to produce melatonin through expression of melatonin-related genes (TPH2, DDC, AANAT, ASMT). Altered melatonin production appears to be feature of children with ASD, including below average physiological levels of melatonin or its metabolites and abnormal coupling of melatonin with the circadian rhythm (107). For example, studies have shown that variations in AMST are associated with decreased melatonin production in ASD (116). Exposure to external environmental factors, such as morning light and external clocks may also contribute to disruptions in sleep cycles. Some individuals with ASD may exhibit variations in light sensitivity, thus leading to a possible misalignment between the circadian phase and light/dark cycle (121). Bidirectional associations between genetic and environmental factors, as well as co-occurring behaviors, are likely to affect underlying biological networks involved in sleep (Figure 2).

#### Summary

Current findings suggest that sleep disorders in children with ASD may be associated with altered circadian rhythms, which may reflect mutations in clock genes and genes involved in melatonin production. Furthermore, sleep disorders in ASD have been associated with behavioral and psychiatric co-morbidities, such as anxiety or ADHD. The directional impact of these underlying mechanisms remains complex when additionally considering the impact of pharmacological interventions, however the broader evidence based suggests co-occurrence of sleep problems with behavioral symptoms, rather than an aetiological mechanism.

### Gastrointestinal Dysfunction

#### Prevalence

Estimates for the prevalence of any gastrointestinal (GI) problem in children with ASD vary between 9 and 70% (7, 14, 122, 123), but may even range to as high as 91% (124). These problems can range from mild gastro-esophageal reflux to more severe symptoms, such as chronic constipation, abdominal pain, and persistent diarrhea (125, 126). The most common of these appears to be chronic constipation, with a median prevalence of 22% (14). Without treatment, these problems can lead to encopresis, delayed continence, pain, and maladaptive daytime behaviors (127–130). Although a subset of children with ASD have GI pathology related directly to ASD, many children also have functional Gl disorders relating to selective eating, medications that have effects on GI motility, and differences in sensory processing. For example, Prosperi et al. (131) found that 27% of children with ASD have problems with food selectivity and that this was frequently associated with GI problems. Despite differences in prevalence between studies, the collective evidence suggests that there is an unusually high prevalence of GI symptoms in children with ASD, implying a possible underlying pathophysiology contributing to both conditions. GI functioning in ASD also encompasses nutrition, including food allergies, metabolic abnormalities, pre-existing nutritional deficiencies, nutrition-related medication side effects, as well as behavioral factors including problematic eating [(132); see Table 5].

**Table 5:** An overview of gastrointestinal (GI) symptoms reported in individuals with ASD.

**Source**	**Sample size**	**Prevalence**	**GI symptoms**
McElhanon et al. (125)	*N* = 15 studies	Higher rates of diarrhea (OR, 3.63), constipation (OR, 3.96), abdominal pain (OR, 2.45).	Diarrhea, constipation, abdomen pain
Mazurek et al. (128)	*N* = 2,973 ASD	24% had at least one type of chronic GI problem.	Constipation, abdominal pain, bloating, diarrhea and/or nausea lasting 3 months or more. Sensory over-responsivity and anxiety were highly associated with GI symptoms.
Mazefsky et al. (129)	*N* = 95 ASD	61% reported at least one GI symptom.	Abdominal pain, not hungry, bloating. Participants with GI problems also had significantly higher levels of affective problems.
Chandler et al. (130)	*N* = 132 ASD, *N* = 81 other developmental conditions, *N* = 82 Controls	46.5% ASD had at least one individual GI symptom, relative to 29.2% other developmental conditions and 21.8% in Controls.	Vomiting, diarrhea, abdominal pain, constipation. No association between GI symptoms and ASD severity.
Prosperi et al. (131)	*N* = 163 ASD	25.8% had at least one severe GI symptom.	Constipated (22.1%), Painful bowel movement (7.4%).

#### Associations With Behavior and Cognition in ASD

It has been speculated that underlying and undiagnosed GI problems may contribute to some of the behavioral difficulties observed in some children with ASD. Chaidez et al. (133) suggested that there is a strong and significant relationship between GI symptoms and increased instances of irritability, social withdrawal and hyperactivity. Furthermore, non-verbal children with ASD often come to clinical attention due to self-injurious behavior and aggression. It is thought that this behavior could be a result of being unable to communicate their pain and/or discomfort effectively. However, there has been limited evidence to suggest a significant association between presence or frequency of GI symptoms and ASD symptom severity over and above those without significant GI complaints (127, 130, 134, 135), although there is some evidence for associations with language impairment (136). Whilst some reports suggest that increased ASD severity is associated with significantly more GI problems (133, 137), a number of studies have failed to support this association (129). In addition, the interplay between GI problems and anxiety symptoms has been supported in both typical and ASD populations. A large-scale study indicated that anxiety, sensory over-responsivity and GI problems are interrelated in children with ASD (128).

#### Treatment Effects

A wide range of diets have been purported to exert some efficacy in alleviating GI symptoms in children with ASD. However, a systematic review found little evidence for the beneficial effect of nutritional supplements or gluten/casein-free diets on ASD symptoms (138). Further to this, the long-term effects of these therapies are not well understood and may have unintentional physical health consequences. For example unconventional food preference may result in reductions in bone cortical thickness in boys with autism, a reduction that is greater for those on casein-free diets (139). However, suboptimal bone development in ASD has also been linked to combinations of a lack of exercise, GI problems, as well as clinically compromised vitamin D and calcium intake due to restrictive diets.

Microbiota transfer therapy has recently been suggested as a potential therapy in ASD to target GI symptoms based on the premise of differences in microbiome composition (132). However, whilst the subject has been widely investigated, findings have been inconclusive and contradictory (see Table 6). For example, in a review of the literature, researchers concluded that inconsistent findings in the field are complicated by use of different methodologies, high incidence of antibiotic use, special diets and/or have repetitive dietary behaviors (144). For example, Kang et al. (142) suggested that GI symptoms in ASD were characterized by less diverse gut microbial composition with findings indicating lower levels of Prevotella, Coproccus, and unclassified Veillonellaceae. It has been argued that a higher diversity of gut bacteria protects the human intestine from stresses such as pathogenic gut microbes and lower diversity in ASD may explain increased risk of GI disturbances. Conversely, Finegold et al. (143) found that there was a significantly higher diversity of bacteria found in feces of participants with ASD. Parracho et al. (124) conducted a study that found that there was a higher incidence of the Clostridium histolyticum group of bacteria. Clostridium histolyticum are known to be toxin-producers and it has been hypothesized that this may contribute toward gut dysfunction with metabolic products having a systemic effect. Based on this evidence, clinical trials have now started investigating whether alteration of microbiota profiles may impact on GI symptoms and ASD behaviors. In a recent open-label trial, microbiota transfer therapy incurred an 80% reduction in GI symptoms, including a significant improvement in symptoms such as constipation and abdominal pain that persisted after 8 weeks, in addition to ASD symptoms (145). These results provide very preliminary evidence that, by targeting the gut ecosystem, potentially both ASD and GI symptoms can be impacted, supporting a potential shared mechanism.

**Table 6:** An overview of studies investigating microbiota composition in ASD.

**References**	**Sample size**	**Methodology**	**Significantly increased in ASD**	**Significantly decreased in ASD**
Williams et al. (140)	*N* = 15 ASD, *N* = 7 Controls; all with GI symptoms	Pyrosequencing and PCR, biopsies from ileal and cecal region	Firmicutes and proteobacteria; Sutterella	Bacteroidetes
Gondalia et al. (141)	*N* = 23 ASD without GI dysfunction, *N* = 28 ASD with GI dysfunction, *N* = 53 siblings	Pyrosequencing of fecal material	No significant difference	No significant difference
Kang et al. (142)	*N* = 20 ASD, *N* = 20 Controls	Pyrosequencing—fecal DNA samples	–	Prevotella, Coproccus and unclassified Veillonellaceae
Parracho et al. (124)	*N* = 58 ASD, *N* = 10 Controls, *N* = 12 Siblings	Fluorescence *in situ* hybridization—fecal material	Clostridium histolyticum group (I & II)	–
Finegold et al. (143)	*N* = 33 ASD, *N* = 8 Controls, *N* = 7 Siblings	Pyrosequencing—fecal microflora	Bacteroidetes, Desulfovibrio, Bacteroides vulgatus, Actinobacterium and Proteobacterium phyla	Firmicutes

#### Aetiological Mechanisms

The underlying mechanism of GI dysfunction and how this relates to the pathophysiology of ASD is still not well understood. One theory in particular has gained particular momentum, that abnormal neurodevelopment in ASD may be caused by increased GI permeability, so-called “leaky gut,” that facilitates the absorption of toxic by-products of incompletely digested proteins [see (146) for review]. The production of metabolites by certain microbiota produces neuroactive compounds, including 5-HT, dopamine and GABA. These can cross the “leaky gut” resulting in the entry of toxins and bacteria into the bloodstream, to influence brain function and the hypothalamic-pituitary-adrenal (HPA) axis [see (147) for review]. This theory has been so influential, that despite limited evidence for efficacy in improving core ASD symptoms, casein and gluten-free diets, as well as dietary supplementations, are increasingly popular alternative treatments pursued by families (148).

**Figure 3:**
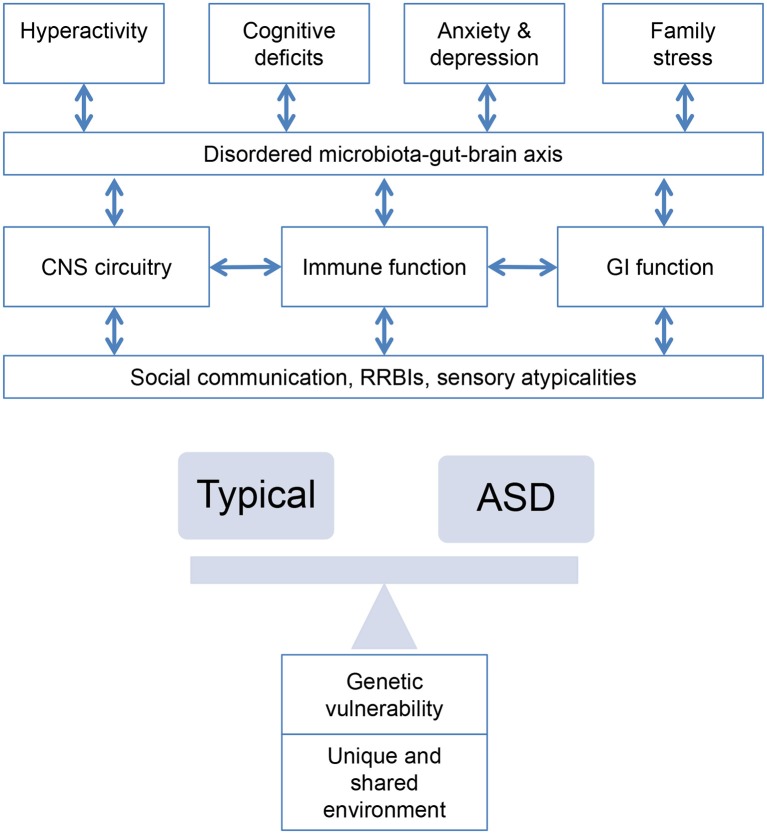
Bidirectional associations between alterations in central nervous system circuitry (epilepsy, sleep), immune system and gastrointestinal function and behaviors characteristic of ASD interacting with genetic and environmental risk.

Serotonin is critical for gut function, with 80–95% of 5-HT receptors localized to the gut and alterations to 5-HT signaling related to many GI disorders (for example, Crohn's disease, ulcerative colitis, irritable bowel syndrome, and chronic constipation). It has been speculated that altered gut 5-HT signaling may be an important contributor to the presence of GI disorders in children with ASD (149, 150); a cascade of events resulting from gut inflammation may lead to reduced levels of brain 5-HT, thereby resulting in mood and cognitive disturbances associated with ASD (149), although this may also be associated with an overall reduced brain availability of 5-HT (151). A well-established connection links the gut to the brain in a bidirectional pathway (see also Figure 3) (152)–autonomic projections from the brain regulate digestive reflexes, signals traveling from the gut signal satiety to the brain, whilst neural signals of stress and anxiety influence gut function and sensitivity. Dysregulation of the HPA axis has been implicated in children with GI problems and emerging evidence suggests a role in ASD (128, 153). This may provide a strong rationale for use of selective serotonin reuptake inhibitors (SSRIs) in the treatment of ASD symptoms by increasing availability of 5-HT, although research to date has established limited efficacy (154). However, if reduced 5-HT results from intestinal inflammation, then possibly targeting 5-HT metabolism by restoring availability of tryptophan for 5-HT synthesis may represent a viable alternate therapeutic mechanism (149).

Increasing evidence suggests GI complications can arise as a result of genetic and environmental risk factors for ASD. For example, variants in the c-Met gene encoding for MET receptor tyrosine kinase are associated with ASD in individuals with co-occurring GI dysfunction (155). The role of MET hypofunction is supported by decreased protein expression in post-mortem brains from autistic individuals compared to typical controls (156). In addition, alterations in the serotonin reuptake transporter (SERT) are implicated in ASD (157) and are likely to disrupt GI serotonin metabolism (158). Expression of the most common SERT variant (Ala56) in mice is associated with ASD-like behaviors [repetitive behaviors, reduced vocalizations, and social contact; (159)], as well as fewer gut neurons, a badly maintained gut lining and slow gut activity (160). Treatment with a 5-HT agonist prevented these GI manifestations (160).

Dysfunctional immune responses (see section Immune Dysfunction), in particular mucosal immune cells, may also have adverse effects on GI functioning in ASD. Endoscopic investigations suggest diffuse inflammation in the intestinal tract of children with ASD (7). There have also been reports of increased gastrointestinal complaints associated with autoimmune responses or a family history of autoimmunity in children in with ASD (161–163), although smaller studies have failed to replicate this increased prevalence (164). For example, increased autoantibodies directed toward central nervous system proteins have been observed in children with ASD and their mothers (165–168). One speculation is that these autoantibodies may signal presence of heightened inflammatory processes or an autoimmune component that could decrease the integrity of the mucosal barrier, or even reflect a downstream effect of previous mucosal infection (123). Notably, these maternal autoantibodies are strongly associated with the functional c-Met C allele associated with susceptibility to ASD and comorbid GI dysfunction (169), forming a promising convergent pathway (see section Immune Dysfunction).

#### Summary

Individuals with ASD are at higher risk of experiencing GI disturbances, with GI problems further linked to increased ASD symptoms. Abnormal neurodevelopment, dysfunctional immune responses and altered serotonergic transmission have been suggested potential mechanisms underlying the overlap between ASD and GI dysfunction.

### Immune Dysfunction

#### Prevalence

The immune system comprises a group of defense mechanisms triggered to protect against disease or illness causing pathogens. Antigens, proteins found on the surface of pathogens, are recognized by a healthy immune system and trigger the production of antibodies that identify and neutralize or remove the antigen. The immune system comprises both innate and adaptive systems. The innate system develops early in fetal development, using genetically encoded receptors and nonspecific mechanisms for defense. In this way, the innate system plays more of a “housekeeping” role. The adaptive system is responsible for threat response, developing a memory of the response for future threats, and does not fully mature until early childhood. This system is more dynamic, responding to potential pathogens and toxins upon exposure. Both systems work together to achieve homeostasis, and importantly, disruption in either can affect neuronal development and functioning (170). Optimal immune functioning is characterized by the homeostatic maintenance of the innate and acquired immune responses, balancing pro- and anti-inflammatory signaling in response to potential pathogenic threats. Aberrant immune functioning can manifest in many ways, such as upregulation of inflammation or immune deficiency that comprises innate host defense mechanisms. Conditions associated with dysfunction of the immune system can include allergies, asthma, and autoimmune disorders.

Prevalence of immune conditions in individuals with ASD varies considerably between studies. Increased rates of food allergies, allergic rhinitis, and atopic dermatitis (i.e., eczema) (133, 171, 172), as well as autoimmune disorders such as psoriasis (4, 173), have been reported in children with ASD in case-control analyses of electronic medical records. In general, asthma is reported at increased frequencies in children with ASD (171, 174), however decreased rates have also been reported (173). Additionally, atopic conditions (asthma, atopic dermatitis, allergic rhinitis, or allergic conjunctivitis) in early childhood is associated with increased rates of later diagnosis of ASD and ADHD, with increased number of atopic conditions associated with stronger likelihood of a later ASD diagnosis (175). Taken together, these studies of medical records or retrospective parental reports support an increased prevalence of specific immune-related conditions, which may constitute an immune-mediated subtype of ASD; see Table 7.

**Table 7:** An overview of immune-mediated conditions reported at increased prevalence in individuals with ASD.

**Study**	**Sample size**	**Conditions**	**Prevalence**
Miyazaki et al. (172)	Meta-analysis; *N* = 10 studies	Atopic dermatitis, asthma, atopic rhinitis, food allergies	Increased rates of asthma (OR 1.66) and atopic rhinitis (OR 1.66) in ASD; no increase in prevalence of food allergies and trend for increase in atopic dermatitis
Chen et al. (171)	*N* = 1,598 ASD, *N* = 6,392 Controls	Type 1 diabetes	Trend for increase in Type 1 diabetes (0.3 vs. 0.1%); OR 4.00
Chen et al. (171)	*N* = 1,598 ASD, *N* = 6,392 Controls	Crohn's disease	Trend for increase in Crohn's disease (1.4 vs. 1.0%); OR 1.46
Chen et al. (171)	*N* = 1,598 ASD, *N* = 6,392 Controls	Urticaria	Increased rates of urticaria (8.4 vs. 6.3%); OR 1.38

#### Associations With Behavior and Cognition in ASD

An extension of this research is the question of whether changes in peripheral cytokine expression are associated with differences in individual behaviors within individuals with ASD. If so, this would then suggest that interventions that target the immune system might have some benefit in improving symptoms. Chemokine levels have been associated with impaired communication, increased parent-reported behavior problems, decreased cognitive and adaptive functions, and worse adaptive behavior (176). Increased levels of proinflammatory cytokines (section Aetiological Mechanisms) have been associated with the severity of ASD in a sample of children with ASD in Egypt (177), whilst decreased levels of an immunosuppressant cytokine, transcription growth factor beta 1, significantly predicts worse behavioral symptoms and lower adaptive levels (178). Al-Ayadhi and Mostafa (179) also found that children with more severe ASD symptoms exhibited greater levels of a proinflammatory cytokine compared to children with more milder symptom presentations. More recently, we observed decreased levels of a range of cytokines associated with symptom severity in children with ASD, with differences in cytokine expressions between male and female children (180). There is also some evidence that different presentations of ASD symptoms may be associated with family histories of immune dysfunction; Molloy and colleagues reported that familial autoimmune thyroid disease was more common in children who regressed compared to those children with an early onset form of ASD (181). Combined, these results suggest a spectrum of altered inflammatory responses associated with differences in symptom profiles within individuals with ASD.

#### Treatment Effects

There is some preliminary evidence for cytokine changes in response to medical treatments in ASD. Choi et al. showed that levels of some cytokines significantly reduced after risperidone treatment in children with ASD, and interleukin 5 was specifically increased in a small sample of treatment responders (182). However, others have not observed changes in cytokine serum levels after risperidone treatment compared to placebo in a much larger sample of children (183). A further study demonstrated that anti-inflammatory agents combined with risperidone had a superior effect in treating symptoms in children, although inflammatory responses were not assessed (184). This latter study implies that modulating the inflammatory response in children with ASD may facilitate the efficacy of treatments such as risperidone. This idea has been supported by some historical and case study accounts of modest reductions in the severity of ASD symptoms when treating gastrointestinal inflammation with corticosteroids or antibiotics (185).

Immune-modulatory agents have also been trialed to specifically target ASD symptoms (186). Significant improvements have been described in open-label investigations of corticosteroids (187) and lenalidomide, an immune-modulatory agent (188), as well as double-blind investigations of celecoxib, an anti-inflammatory drug (189). However, varying clinical efficacy and limited randomized double-blind investigations, combined with serious side effects noted in corticosteroids (189), indicates that this area requires further investigation.

#### Aetiological Mechanisms

The mechanisms by which dysfunctional immune systems may contribute etiologically to ASD is an area of active investigation (190). Here, we briefly outline the main streams of research [see (191) for extensive recent review on aetiological mechanisms of immune dysfunction in ASD]. Several reports of associations between ASD and family history of autoimmune or immune-mediated disorders have emerged (161, 192, 193). Several high confidence genes identified for ASD converge on pathways important in synapse formation, neuronal migration, and immune function (194, 195). Of relevance here, restriction of HLA genes has been reported as conferring a greater risk for the development of ASD, involved in immune function and also associated with risk for autoimmune conditions (196–199). Located within the large genome regions known as the major histocompatibility complex (MHC) on chromosome 6, several HLA haplotypes appear to be more frequent in children with ASD (200, 201). Some failures to replicate this have suggested that possible genetic differences may lie more generally within this MHC region rather than specifically confined to the HLA genes only (202, 203). For example, the gene coding for the complement protein C4 located in the MHC region is important for innate immunity. Deficiencies in the C4B allele, as well as several other complement proteins, may be differentially produced in some individuals with ASD (204–208). In addition, the MET receptor tyrosine kinase has been associated with ASD (209), which is implicated in both neurodevelopment and immune function.

In addition to examining diagnosed immune conditions, significant research has focused on measurements of cytokine signaling profiles as indicators of broader changes in inflammatory processes in individuals with ASD (17). Cytokines are proteins produced and expressed by neurons that regulate immune responses, including hematopoiesis, inflammation, and immune cell proliferation and differentiation (210). Some cytokines act to make disease worse (proinflammatory) whereas others reduce inflammation and promote healing by suppressing the activity of proinflammatory cytokines (anti-inflammatory). Cytokines also play a role in normal neurodevelopment, including the processes of neuronal migration and synaptic plasticity (211). These processes are tightly regulated and a dysregulation in the balance of signals mediated by cytokines can have a variety of detrimental effects that contribute to changes in neurodevelopment and behavior.

A recent meta-analysis of cytokine levels derived from plasma and serum in unmedicated individuals with ASD (mostly children) found an overall abnormal cytokine profile, characterized by elevations in proinflammatory cytokines and reduced levels of anti-inflammatory cytokines (17). This suggests that some individuals with ASD may exhibit a heightened inflammatory state and altered cytokine profile, observed in peripheral tissues, suggestive of broader immune system dysregulation in ASD. While elevated levels of inflammatory cytokines observed in the central nervous system of individuals with ASD may reflect inflammatory processes that modulate neuronal function and change behavior (79, 212), altered cytokines in peripheral tissues indicate more widespread inflammatory involvement (213–216). This meta-analysis also observed significant heterogeneity between studies, likely reflecting important methodological differences between studies and the prevalent use of siblings as control subjects for analysis, who are at higher risk of exhibiting broader ASD symptoms themselves (217) or having similar immune system profiles (218).

One well-researched area has explored early maternal infection and inflammation during pregnancy and later risk for an ASD diagnosis (219–225). Pregnancy requires a complex and dynamic response from the maternal immune system to protect the mother from pathogens or infections but to also support the fetal tissue that contains many “non-self” antigens from the father to promote fetal health and development. As this is a complex regulatory system to maintain such balance, this period represents an extremely vulnerable period for both mother and the developing fetus. Disturbances in immune regulation during this period has been well-established to provide a substantial risk factor for alterations in neurodevelopment (226). Incidence of maternal viral and bacterial infections has been proposed as a risk factor for the development of ASD (220, 227–229). Perturbation of the maternal immune system may modify either the placenta or the fetal brain to then later neurodevelopment (230, 231), of which the cytokine IL-6 appears to play a major role (231, 232). Involvement of the maternal immune system during pregnancy does not just appear to influence neurodevelopment, but may also modify ongoing immune dysfunction in offspring (233–236) and later symptom presentation (237). The maternal immune environment has also been proposed as a key factor influencing the increased risk of ASD diagnoses in children born very preterm (226).

#### Summary

Disruptions in both innate and adaptive immunity have clear consequences for neurodevelopment, with the cumulative evidence suggestive of a disrupted immune profile in for some individuals with ASD as well as links to the early maternal pregnancy environment in shaping later immune profiles and neurodevelopment. There evidence to suggest a direct association between perturbed immune profiles and impact on subsequent behavioral and symptomatic profiles, with limited studies for potential changes in immune profiles in response to treatment or changes in immune functioning through anti-inflammatory markers to facilitate reductions in core ASD symptoms. Taken together, this evidence suggests that an immune-mediated subtype of ASD may be amenable to specific, targeted and/or personalized treatments based on individual immune profiles.

## Discussion

### Mechanisms of Associations Between Medical Comorbidities and ASD

This review highlights that the interactions between observed comorbid medical conditions and physiological abnormalities in children with ASD are complex. Disorders of GI function and seizures appear to be parallel comorbid conditions, with possible common aetiological mechanisms resulting from yet unknown neurological causes. Sleep disorders, however, are likely to be a consequence of ASD symptoms or may be, in turn, associated with GI symptoms or other comorbidities that can cause sleep disturbances. Whilst the evidence discussed suggests a causative role for metabolic and immunological pathways for some individuals later diagnosed with ASD, evidence for these remains preliminary and based on group level findings, rather than evidence from prospective and longitudinal observations. What is clear, however, is that these systems and pathways do not work in isolation from each other; rather, complex interactions imply dysregulation in any one system may cause a cascade of events cumulating in a cluster of symptoms associated with ASD (Figure 3). Because these systems are very complex, and no perturbations in any one appear to be common across individuals with ASD, it is likely that the heterogeneity in ASD reflects the potential myriad of different disturbances along any one of these pathways. Further complicating this model is the high likelihood that any given individual with ASD may also present with multiple medical comorbidities or elevated abnormalities, which also interact with co-occurring behavioral comorbidities, such as hyperactivity and anxiety, and developmental delay (238). Thus, attempts to subgroup individuals based on medical conditions alone may be complicated by the prospects of individuals belonging to multiple subgroups. Importantly, identification of shared or distinct biochemical or neurocognitive mechanisms will be key in elucidating causal pathways.

### Integrating Mechanistic Models of ASD: Implications for Etiology and Treatment

As discussed in each section, complicating interactions between these systems suggest a greater likelihood for individuals with alterations in one system to have alterations across multiple systems. Still, the overlap between medical and behavioral features associated with ASD may point toward convergent platforms for a final common pathway to ASD across varied causes, with implications for targeted treatment. For example, children who have sleep problems and ASD are two times more likely to have GI issues and seizures (6).

Review of the proposed aetiological mechanisms underlying the comorbidity between these medical comorbidities and ASD allows some degree of integration. Processes with shared involvement in ASD and multiple medical disorders include gene transcriptional regulation; cellular growth and proliferation; and synapse development, stability and function. Importantly, the potential role of the microbiota-gut-brain axis in multiple elements of medical comorbidity has been implicated (Figure 3), whereby short-chain fatty acids can cross the blood-brain barrier and enter the brain (section Gastrointestinal Dysfunction, Table 6), gut microbiota modulate the immune response by stimulating secretion of cytokines and microbiota can deliver signals to the brain via the vagus nerve [see (239) for review]. Beneficial therapeutic effects may be afforded by focusing on the microbiota-gut-brain axis, although more systematic study of its role in ASD is required.

Associations between sleep and other disorders are also apparent; while classically functioning to regulate circadian and seasonal rhythms, melatonin additionally affects cardiovascular and immune systems, regulates body fat mass, insulin secretion, and metabolism of glucose and lipids, as a close derivative of serotonin (5-HT). Due to these additional functions, emerging research has highlighted a role for melatonin administration in humans to attenuate metabolic symptoms induced by antipsychotic use (240) and to potentially improve GI functioning via effects on intestinal permeability (241). Given additional physiological functions of melatonin, effective supplementation may have secondary effects in improving GI functioning in ASD; however there has not been any systematic investigation into this as yet.

Similarly, comorbid medical conditions and associated medication use may result in sleep disturbance; for example, physical symptoms such as abdominal pain in some GI disorders, or medications prescribed for seizures can cause difficulty sleeping. Children with epilepsy also exhibit significant alterations in sleep latency, sleep efficiency, and number of awakenings (242), and epilepsy may be characterized by seizures during sleep (e.g., Landau-Kleffner). It is therefore of critical importance to determine whether sleep problems are better explained by associated medical conditions which should be treated rather than attempting to address sleep difficulties alone (100).

Finally, alterations in immune system profiles, dysregulated gastrointestinal symptoms and disordered sleep patterns clearly impact upon behavioral profiles, including elevated anxiety, increased social and communication difficulties, reduced adaptive functioning, and increased maladaptive behaviors. Recent meta-analytic evidence confirms an association between sleep disturbances and increased inflammation, suggesting that improvements in one system could potentially positively impact upon other regulatory systems (243). Generating evidence for such hypotheses require large and detailed biological datasets from children with ASD across their developmental course [e.g., (244–246).

A key aim for future research will be to examine the interactions between these systems using established biomarkers along their pathways within ASD, rather than examining each in isolation (Box 1). Possible networks of disturbances may then be mapped out to determine potential subgroups of individuals with ASD classified by patterns of abnormal biological profiles. A second question of interest will be how modulation of these biological substrates through targeted pharmacological interventions may affect core ASD symptoms (247). Initial promising findings in small sampled trials suggest some efficacy for such approaches. However, if subgroups of individuals with specific biological profiles exist along the spectrum, then interventions must be appropriately targeted for individuals with certain profiles, rather than a universal approach. Lastly, the true efficacy of such targeted interventions will lie in the use of robust and sensitive biomarkers to determine treatment response, rather than a sole reliance on parent-reported and observational outcomes.

Box 1 **Future directions and common themes.**

- Using expertise from a diverse range of disciplines—genetics, neuroscience, biochemistry and developmental psychology—to disentangle biological mechanisms underpinning medical comorbidity.
- Improved understanding of the link between comorbid medical conditions and co-occurring mental health problems and psychopathology in ASD.
- Understanding the longitudinal course of the full range of medical comorbidities in ASD.
- Treatment and intervention studies to systematically assess whether treating medical issues has additional positive effects on other medical issues (e.g., sleep) or on behavioral domains.

An important consideration is the time at which symptoms or markers are assessed. As a developmental condition, the symptoms of ASD change over the course of the child's lifespan, and there is increasing acknowledgment for the importance in understanding an individual's trajectory over time to grasp the full complexity of the heterogeneous symptoms presentations within ASD (248). Current physiological states are dependent on history of previously received interventions and past characteristics, such as regression, that may later affect developmental trajectories. In addition, there are likely to be sensitive periods within development where particular comorbidities may be more apparent or exhibit higher risk; for example, sensitive periods for prevalence of epilepsy-related disorders in childhood (249). Prospective longitudinal studies are required to systematically measure medical disorders and biomarkers within cohorts of individuals diagnosed or with clinical risk indicators of ASD. This may then provide sufficient power to identify subgroups of children with different clusters of symptoms that converge on similar pathways. Likewise, a developmental perspective will provide insight on the nature of the association with health conditions as a resulting or co-occurring model.

### Clinical Implications

Assessment of co-occurring medical problems is of critical importance in the initial diagnostic procedure for individuals with ASD as well as in ongoing treatment management. As part of a multidisciplinary approach, systematic and evidence-based screening should be recommended at the time of diagnostic evaluations as well as during ongoing health monitoring. A key conclusion from this review is the importance of monitoring and maintaining general health and wellbeing in individuals with ASD. Although many of the medical conditions or abnormalities may not be present in many individuals with ASD, the overall increased risk of all-cause mortality in this population indicates a need for increased vigilance. For example, comorbid epilepsy, particularly accompanied by intellectual disability, is associated with an increased mortality risk; thus, there is a significant imperative for clinicians and parents/careers to provide additional care and monitoring around overall health and wellbeing for these individuals to potentially attenuate this risk. Cumulative evidence highlights increased prevalence of obesity and poor diet in individuals with ASD, and decreased use of general health services, such as for oral health, due to barriers to accessing health care that supports individuals with special needs. There is also a critical need for healthcare services to provide supportive and accessible environments for individuals with ASD.

The burden of such medical conditions in increasing mortality is likely compounded by impaired communication and increased sensory sensitivities that present a significant barrier to the delivery of health care services (both preventative and targeted treatment). A growing area of research has therefore focused on the necessity for effective and targeted health care services that are designed for ASD, as well as identifying potential barriers to access and use (250). Appropriately delivered health care services that can manage difficulties in communication and sensory sensitivities, particularly those that are pre-emptive, are likely to have benefits beyond just medical treatment but may drastically improve the quality of life for both the individual and their caregiver(s) and potentially decrease long-term financial burden. Despite higher health care utilization in this population, the decreased ability for the health care system to effectively manage behavioral symptoms, impaired communication, and specific sensory issues is a significant barrier for most preventative health care, especially for those on the severe end of the spectrum.

## Conclusion

The presence of comorbid medical conditions in ASD highlights the vast heterogeneity within the disorder. Using a model-based approach to understand the interacting systems potentially involved in the etiology and symptoms of ASD may lead to hypothesis generation and potential avenues for clinical trials. We require a better understanding of how variability in these systems results in similar or different functional profiles in ASD, and more integrative studies that consider the interaction between systems and the environment to produce behaviors characteristic of ASD. Such an approach can then lend hope to more specific biomedical treatments aimed at targeted these biomedical abnormalities and improving core and associated symptoms in ASD, ultimately to improve long-term outcomes.

## Author Contributions

CT and GA led in the conception and design of the review. CT, AR, and GA drafted the paper. AW provided substantial revision.

### Conflict of Interest Statement

The authors declare that the research was conducted in the absence of any commercial or financial relationships that could be construed as a potential conflict of interest.
