# Corrigendum: Characterizing the Interplay Between Autism Spectrum Disorder and Comorbid Medical Conditions: An Integrative Review

**DOI:** 10.3389/fpsyt.2019.00438

**Published:** 2019-06-27

**Authors:** Charlotte Tye, Abigail Runicles, Andrew J.O. Whitehouse, Gail A. Alvares

**Affiliations:** ^1^Child & Adolescent Psychiatry, Institute of Psychiatry, Psychology & Neuroscience, King’s College London, London, United Kingdom; ^2^Telethon Kids Institute, The University of Western Australia, Perth, WA, Australia; ^3^Cooperative Research Centre for Living with Autism (Autism CRC), Brisbane, QLD, Australia

**Keywords:** autism spectrum disorder, comorbidity, epilepsy, gastrointestinal disorders, immune function, sleep

There was an omission in the **Funding** statement. The grant number for “Medical Research Council” is “G9817803”.

The authors apologize for this error and state that this does not change the scientific conclusions of the article in any way. The original article has been updated.

